# Comparison of Cumulative Live Birth Rate Between Aged PCOS Women and Controls in IVF/ICSI Cycles

**DOI:** 10.3389/fendo.2021.724333

**Published:** 2021-09-28

**Authors:** Zhuoyao Mai, Manlin Liu, Ping Pan, Lin Li, Jia Huang, Xiaoli Chen, Dongzi Yang

**Affiliations:** Department of Obstetrics and Gynecology, Sun Yat-Sen Memorial Hospital of Sun Yat-Sen University, Guangzhou, China

**Keywords:** ageing, *in vitro* fertilization (IVF), polycystic ovary syndrome (PCOS), propensity score matching (PSM), cumulative live birth rate

## Abstract

The present study aimed to assess whether women with polycystic ovarian syndrome (PCOS) ≥35 years age undergoing *in vitro* fertilization (IVF)/intracytoplasmic sperm injection (ICSI) cycles experienced a higher cumulative live birth rate (CLBR) over a two-year period compared with age- and body mass index (BMI)-matched patients with tubal factor infertility. Through propensity score matching (PSM) approach, the authors retrospectively analyzed the IVF/ICSI outcomes of 263 PCOS patients (35-46 years of age [mean, 37 years]) and 526 age- and BMI-matched tubal factor controls two years after oocyte retrieval. Multivariate regression analysis was performed to explore factors influencing cumulative live birth. Women with PCOS exhibited better ovarian reserve and response, and higher CLBR in two years compared with age- and BMI-matched controls (CLBR: 55.51% in PCOS *vs*. 38.02% in control, p<0.001). Multivariate logistic regression analysis revealed that the number of transferable embryos and antral follicle counts were both significant independent factors predicting cumulative live birth after adjusting for female age, female body mass index, percentage of transferred blastocysts, number of embryos transferred per embryo-transfer cycle, diagnosis of PCOS and freeze-all cycles (p<0.001, p=0.045). Women with PCOS ≥ 35 years of age demonstrated a higher CLBR over two years compared with age- and BMI-matched controls. This could be explained by favorable oocyte reserve and more available embryos compared with controls, which overcome the compromised oocyte quality in aged PCOS patients.

## 1 Introduction

Polycystic ovarian syndrome (PCOS) is a common endocrine disorder that affects approximately 6%-10% of reproductive-age women (1). Ovulation abnormalities cause infertility in women with PCOS, while other factors, including metabolic morbidities, obesity, hyperandrogenism and high luteinizing hormone(LH) levels, increase the complexity of the problem (2–4). Modern society has witnessed women postponing marriage and childbearing. However, the fecundity of women declines with age. Although assisted reproductive technology(ART) has helped to increase pregnancy rates (5), continuous attrition of oocytes, compromised oocyte quality and cumulative adverse reproductive tract factors inevitably limit the success of ART in women ≥ 35 years of age (3). Therefore, the fecundity of women with PCOS ≥ 35 years of age undergoing IVF is a notable concern.

Despite its heterogeneity in phenotype and morbidity, women with PCOS appear to have an advantage in ovarian reserve. The median density of small preantral follicles has been reported to be six-fold greater in biopsies from polycystic ovaries in anovulatory women than in normal ovaries (6). Anti-Müllerian hormone (AMH) is mainly secreted by the granulosa cells of preantral and small antral follicles 5-8mm in diameter (7). Because the concentration of serum AMH is proportional to the number of developing follicles in the ovaries, it is considered to be a marker of ovarian reserve and ageing of ovaries (8). Serum AMH levels and antral follicle counts (AFCs) in women with PCOS are significantly higher than those of age-matched controls (9). These remain until the age at onset of perimenopause, in addition to lower serum follicle-stimulating hormone (FSH) levels compared with controls (10).

Abundant ovarian reserve in women with PCOS has a corresponding ovarian response in controlled ovarian hyperstimulation during *in vitro* fertilization (IVF) cycles (11). Whether the advantages of ovarian reserve in the PCOS population leads to success in live births in aged individuals is controversial. A meta-analysis concluded that women with PCOS achieved similar clinical pregnancy and live birth rate (LBR) per started IVF cycle as normo-ovulatory women (11). A retrospective cohort study reported that oocytes retrieved and LBR remained stable in the PCOS group, but decreased in age-matched controls across the age range of 22-41 years (12). A large-scale study reported that women with PCOS had an increased number of oocytes retrieved, clinical pregnancy rate and LBR until 40 years of age compared with controls, although similar declines in clinical pregnancy rate and LBR with age were detected in both groups (13).

These studies, however, focused only on fresh cycles. With the development of embryo cryopreservation technology, embryos can be well-preserved, thawed and transferred to the uterus after ovary recovery and previous preparation of the endometrium. Therefore, consideration of an overall LBR in fresh and frozen cycles of embryo transfer (ET) after each oocyte retrieval is significant and comprehensive, especially in women ≥ 35 years of age in their eager desire to have children. Studies investigating cumulative live births in PCOS patients ≥35 years of age undergoing IVF-ET are scarce. One comparative analysis reported that women with isolated polycystic ovary morphology (PCOM), instead of PCOS, had a higher cumulative live birth rate (CLBR) than controls (14). Favorable ovarian reserve in PCOS individuals ≥ 35 years of age is obvious. However, whether this advantage leads to a better LBR and CLBR remains uncertain. Therefore we used propensity score matching (PSM) approach, to compare the LBRs and CLBRs of IVF/ICSI cycles within two years of follow-up, in infertile PCOS patients ≥ 35 years of age and age- and body mass index (BMI)-matched controls.

## 2 Material and Methods

### 2.1 Study Population

Patients ≥ 35 years of age who underwent undergoing their first IVF/ICSI cycle between January 1, 2014 and August 31, 2017, at the Center for Reproductive Medicine in Sun Yat-Sen Memorial Hospital (Guangzhou, Guangdong Province, China) were retrospectively identified in the institutional database. A total of 263 patients with PCOS and 1789 with tubal factors as the single underlying cause of infertility were included. The diagnosis of PCOS was based on the 2003 Rotterdam consensus (15), with at least two out of the following three conditions and exclusion of other etiologies (congenital adrenal hyperplasia, androgen-secreting tumors and Cushing’s syndrome): oligo- or anovulation; clinical and/or biochemical signs of hyperandrogenism; and polycystic ovaries. Individuals with a history of ovarian surgery, genital tumors, other endocrine disorders, endometriosis or uterine malformations were excluded.

The initial control group (n=1789) differed greatly from patients with PCOS in both age and BMI (age: median, 36 years [interquartile rage, 35,38] in PCOS group *vs*. median, 38[interquartile rage, 36,40] in control group, p<0.001). To reduce confounding bias between the groups and to attain a convincing result, a PSM approach was used. Propensity scores were calculated using logistic regression based on female age and BMI using the nearest neighbor random matching algorithm. PSM was performed at a ratio of 1:2. Demographic data, cycle information, reproductive outcomes and clinical features were compared between the groups.

This study was approved by the Human Research and Ethics Committee of the Sun Yat-sen Memorial Hospital. Written informed consent was obtained for IVF/ICSI process.

### 2.2 Stimulation Protocols for IVF

Ovarian stimulation was performed using gonadotropin (Gn)-releasing hormone (GnRH) agonist long protocol or GnRH antagonist protocol. Ovarian stimulation and IVF-ET were performed as previously described (16).

#### 2.2.1 GnRH Agonist Long Protocol

Pituitary downregulation in the midluteal phase of the menstrual cycle was achieved by intramuscular (i.m.) injection of 0.75-1.25 mg long-acting GnRHa (IPSEN, Paris, France), or daily administration (i.m.) of 0.05-0.1 mg short-acting GnRHa (IPSEN, Paris, France) until the day of human chorionic gonadotropin (HCG) administration. Two to three weeks later, vaginal ultrasound and blood hormone tests (FSH, LH, estradiol [E2]) were performed to evaluate the complete downregulation of the pituitary gland. Once these results were confirmed, controlled ovarian hyperstimulation (COH) was initiated with Gn 75U - 300 U/day, while dosage was determined according to individual BMI, basal hormone levels and AFC. Evaluation of follicular growth was performed using vaginal ultrasound and blood hormone tests every three to five days, and adjustment of Gn dosage was performed when necessary. HCG (Livon, China) 4000 U to 10,000 U was administered to induce oocyte maturation, when at least one follicle ≥ 18mm or three follicles ≥17 mm in diameter. Transvaginal oocyte retrieval was performed 36 to 38 h after the HCG trigger, under the guidance of vaginal ultrasound.

#### 2.2.2 GnRH Antagonist Protocol

After evaluation of follicular condition according to blood hormone tests (FSH, LH, E2) and vaginal ultrasound, Gn 75 U to 300  U/day was administered to stimulate follicular growth. Follicular development was evaluated using vaginal ultrasound and blood hormone tests every three to five days, and Gn dosage adjusted when necessary. GnRH antagonist was added on the fifth to seventh day after Gn stimulation began (i.e., fixed protocol), or according to the size of the dominant follicle and LH level (i.e., flexible protocol). HCG (Livon, China) 4000 U to 10,000 U or GnRHa (IPSEN) 0.2 mg was administered to induce oocyte maturation, when at least one follicle ≥18 mm or three follicles ≥ 17 mm in diameter. Oocyte recovery was performed 36 - 38 h after the trigger.

### 2.3 Embryo Scoring and Embryo Transfer

Embryos were scored according to morphology assessment described by the Istanbul Consensus. Cleavage stage embryos scored grade 1 and day 5 blastocysts scored grade 3 or grade 4 were considered to be good-quality embryos.

In fresh cycles, embryos were cultured *in-vitro* until the third to fifth day after oocyte retrieval, and transferred to the uterus, with a maximum of three embryos. Corpus luteal support was provided on the day of oocyte retrieval, with progesterone injections (i.m., 60mg/day) or progesterone capsules (600 mg/day trans-vaginal), until the pregnancy test. If patients exhibited evidence of ovarian hyperstimulation syndrome (OHSS) or were at risk of OHSS (number of oocytes retrieved > 25, or E2 > 4800 ng/ml on HCG day), ET would be canceled and good-quality embryos would be cryopreserved.

In frozen ET (FET) cycles, a maximum of three embryos were placed in the uterus on the third or fifth day after ovulation in natural cycles(NC) or induced ovulation (IO) cycles, or after the beginning of progesterone administration in artificial cycles. Corpus luteal support was administered since ovulation (in NC and IO), or when the endometrium was prepared in artificial cycles, with progesterone injections (i.m., 60 mg/day) or progesterone capsules (600 mg/day trans-vaginal) until pregnancy testing.

### 2.4 Measurement

A pregnancy test was performed 14 days after ET, with a serumβ- HCG level >5 IU/L considered to be positive. Subsequently, clinical pregnancy was defined as a gestational sac detected on ultrasound four weeks after embryo transfer. Cumulative live births in this study were calculated until two years after ovum pick up, or exhaustion of preserved embryos before two years, including both fresh ET cycles and subsequent FET cycles.

### 2.5 Statistical Analysis

Data analyses were performed using Statistical Package for Social Sciences version 24.0 (SPSS IBM Corporation, Armonk, NY, USA). In the present study, non-normally distributed continuous variables are expressed as median (25th quartile, 75th quartile, i.e., interquartile range [IQR]); categorical variables are described as numbers and percentages. Data were compared using the Wilcoxon rank sum test, t-test or chi-squared test, where appropriate. Statistical significance was defined as a two-tailed p-value of 0.05. Logistic regression analysis was performed to examine factors predicting CLBR in two years.

## 3 Results

After PSM at a ratio of 1:2, 263 PCOS patients and 526 tubal factor infertile patients were included in the final analysis.

Data reported in **Table 1** show that female age, BMI and male age were comparable between the groups. The PCOS group had a significantly longer duration of infertility (7 years versus *vs*. 4 years, p<0.001), but better ovarian reserve, with higher AFC (26 *vs*. 12, p<0.001) and higher serum AMH level (7.48 *vs*. 2.89 ng/ml, p<0.001) compared with the control group. Infertility type differed between the groups (percentage of primary infertility: 48.67% in the PCOS group *vs*. 23.95% in the control group, p<0.001). During controlled ovarian stimulation, PCOS patients received a lower initiation Gn dose (150 U *vs*. 225 U, p<0.001), but they had higher estrogen levels on HCG days (3328 *vs*. 2725 ng/ml, p<0.001) than controls. Stimulation protocols differed significantly between the PCOS and control groups: GnRH antagonist protocol, 109/263 (41.44%) *vs*. 110/526 (20.91%), respectively; long GnRH agonist protocol, 154/263 (58.56%) *vs*. 416/526 (79.09%), respectively (p<0.001). Ovarian response was better in the PCOS group, with a higher number of retrieved oocytes (12 *vs*. 10, p<0.001). The number of patients who withheld fresh ET due to the risk of OHSS was 44 (16.73%) in the PCOS group and 49 (9.32%) in the control group. Other patients canceled fresh ET due to fever or personal reasons.

**Table 1:** Baseline characteristics and ovarian stimulation of PCOS women aged 35 or older and age-, BMI-matched tubal factor controls in IVF/ICSI cycles.

	Control	PCOS	P value
n	526	263	
Female age, y	37 (36,38)	37 (36,38)	0.447
Male age, y	39 (36,42)	39 (36,41)	0.152
Body mass index, kg/m^2^	23.2 (21.0,25.3)	23.2 (21.0,26.1)	0.827
Infertile duration, y	4 (2,8)	7 (4,9)	<0.001
Type of infertility			<0.001
Primary infertility,%	23.95 (126/526)	48.67 (128/263)	
Secondary infertility,%	76.05 (400/526)	51.33 (135/263)	
Antral follicle count, n	12 (8,18)	26 (23,31)	<0.001
Serum AMH, ng/ml	2.89 (1.45,4.96)	7.48 (5.11,11.02)	<0.001
Gn initiation dose, IU	225 (150,250)	150 (113,200)	<0.001
Total Gn dose, IU	2475 (1919,3000)	1800 (1463,2475)	<0.001
Gn treatment, days	11.0 (10.0,13.0)	11.0 (10.0,13.0)	0.975
Protocol			<0.001
Long GnRH agonist protocol,%	79.09 (416/526)	58.56 (154/263)	
GnRH antagonist protocol,%	20.91 (110/526)	41.44 (109/263)	
Estradiol on HCG day, ng/ml	2725 (1554,4232)	3328 (2103,4840)	<0.001
Number of retrieved oocytes	10 (7,15)	12 (9,17)	<0.001
Number of transferable embryos	6 (3,9)	6 (4,10)	0.002

PCOS, Polycystic ovary syndrome; IVF, in vitro fertilization; ICSI, intracytoplasmic sperm injection; AMH, anti-Müllerian hormone; Gn, gonadotropin; GnRH, gonadotropin-releasing hormone; hCG, human chorionic gonadotropin.

Values are described as median (25th quartile,75th quartile) or percentage (number/total number).

The PCOS group underwent 189 fresh ET cycles (71.86%) with 386 embryos. Among these, 26 cycles (13.76%) were performed with one embryo, 129 cycles (68.25%) with two, and 34 cycles (17.99%) with three, as is shown in **Table 2**. The control group underwent 369 fresh ET cycles (71.15%) with 710 embryos. Among these, 77 cycles (20.87%) were performed with one embryo, 243 cycles (65.85%) with two, and 49 cycles (13.28%) with three. The proportions of cycles with different numbers of transferred embryos were similar between the PCOS and control groups (p=0.067). Both average number of embryos transferred and percentage of transferred blastocysts were higher in the PCOS group than in control group in fresh ET cycles (mean number of embryos transferred: 2.04 ± 0.56 *vs*. 1.92 ± 0.58 respectively, p=0.022; percentage of transferred blastocyst: 55/386 [14.25%] in PCOS group *vs*. 34/710[4.79%] in control group, p<0.001). Pregnancy outcomes are shown in **Table 2**. Patients with PCOS exhibits higher clinical pregnancy rate and live birth rate in fresh ET cycles than controls (clinical pregnancy rate per ET: 106/189[56.08%] in PCOS group *vs*. 129/369[34.96%] in control group, p<0.001; live birth rate per ET: 84/189[44.44%] *vs*. 88/369[23.89%], p<0.001). Abortion rates of fresh ET cycles are similar between groups (22/106[20.75%] in PCOS group *vs*. 40/129[31.01%] in control group, p=0.076).

**Table 2:** Pregnancy outcomes of PCOS women aged 35 or older and age-, BMI-matched tubal factor controls in IVF/ICSI cycles.

	Control	PCOS	P value
n	526	263	
Fresh cycle outcomes			
ET cancel for OHSS risk,%	9.32 (49/526)	16.73 (44/263)	0.003
Average number of embryos transferred, n	1.92 ± 0.58	2.04 ± 0.56	0.022
Cycles distribution			0.067
with 1 embryo transferred,%	20.87 (77/369)	13.76 (26/189)	
with 2 embryos transferred,%	65.85 (243/369)	68.25 (129/189)	
with 3 embryos transferred,%	13.28 (49/369)	17.99 (34/189)	
Percentage of transferred blastocyst,%	4.79 (34/710)	14.25 (55/386)	<0.001
Pregnancy rate per cycle start,%	24.52 (129/526)	40.30 (106/263)	<0.001
Live birth rate per cycle start,%	16.73 (88/526)	31.94 (84/263)	<0.001
Pregnancy rate per ET,%	34.96 (129/369)	56.08 (106/189)	<0.001
Live birth rate per ET,%	23.89 (88/369)	44.44 (84/189)	<0.001
Miscarriage rate,%	31.01 (40/129)	20.75 (22/106)	0.076
Frozen-thawed cycle outcomes			
Average number of embryos transferred, n	3.25 ± 1.89	2.98 ± 1.93	0.054
Cycles distribution			0.046
with 1 embryo transferred,%	23.30 (130/558)	18.14 (37/204)	
with 2 embryos transferred,%	52.15 (291/558)	62.25 (127/204)	
with 3 embryos transferred,%	24.55 (137/558)	19.61 (40/204)	
Percentage of transferred blastocyst,%	34.46 (387/1123)	40.15 (165/411)	0.040
Pregnancy rate per ET,%	30.29 (169/558)	42.16 (86/204)	0.020
Live birth rate per ET,%	20.79 (116/558)	31.37 (64/204)	0.002
Miscarriage rate,%	31.36 (53/169)	20.93 (18/86)	0.079
Cumulative outcomes			
Number of embryos transferred per ET, n	2.01 ± 0.56	2.02 ± 0.51	0.686
Percentage of transferred blastocyst,%	22.97 (421/1833)	27.60 (220/797)	0.011
Cumulative pregnancy rate,%	51.90 (273/526)	66.92 (176/263)	<0.001
Cumulative live birth rate,%	38.02 (200/526)	55.51 (146/263)	<0.001

PCOS, Polycystic ovary syndrome; IVF, in vitro fertilization; ICSI, intracytoplasmic sperm injection ET, embryo transfer; FET, frozen embryo transfer.

Values are described as mean ± standard deviation or number/total number (percentage).

In the PCOS group, 204 frozen-thawed ET cycles were performed with 411 embryos. Among these, proportions of cycles with one, two or three embryos transferred were 18.14%, 62.25% and 19.61% respectively, as shown in **Table 2**. The control group underwent 558 frozen-thawed ET cycles with 1123 embryos in total. The proportions of cycles with one, two or three embryos transferred were 23.30%, 52.15% and 24.55% respectively. The control group had a slightly higher average number of embryos transferred than the PCOS group in FET cycles (3.25 ± 1.89 *vs*. 2.98 ± 1.93, respectively); however, the difference was not statistically significant (p=0.054). The PCOS group had a higher percentage of transferred blastocysts than the control group in FET cycles (165/411 [40.15%] *vs*. 387/1123 [34.46%], respectively, p=0.040). Significant differences were detected in the clinical pregnancy and LBR of FET (clinical pregnancy rate per FET: 86/204 [42.16%] in the PCOS group *vs*. 169/558 [30.29%] in the control group, p=0.020; LBR per ET: 64/204 [31.37%] *vs*. 116/558 [20.79%], p=0.002). Abortion rates in FET cycles were comparable between the PCOS and control groups (18/86 [20.93%] *vs*. 53/169 [31.36%], respectively, p=0.079).

Considering both fresh and FET cycles, the mean number of embryos transferred per ET were similar between the two groups (2.01 ± 0.56 in the control group *vs*. 2.02 ± 0.51 in the PCOS group, p=0.686). The percentage of transferred blastocysts including fresh and FET cycles, was still higher in the PCOS group than in the control group (27.60% *vs*. 22.97%, respectively, p=0.011). Overall, CLBR in two years in patients with PCOS was approximately 50% higher than that in patients with tubal factor infertility (146/263 [55.51%] *vs*. 200/526 [38.02%], respectively, p<0.001).

In the control group, two (0.38%) patients had triplet pregnancies, one of whom gave birth to twins after reduction, and the other experienced spontaneous abortion. Fifty-six (10.64%) patients had twin pregnancies in the control group, thirty-six of whom gave birth to twins, eight experienced miscarriage, and the remaining twelve eventually had single deliveries (two received reduction and ten experienced spontaneous abortion of one fetus). In the PCOS group, all four (1.52%) patients with triplet pregnancies received reduction and achieved one single live birth and three twin live births. Fifty (19.01%) patients had twin pregnancies in the PCOS group, twenty-six of whom gave birth to twins, ten experienced miscarriage and the remaining fourteen had single delivery (four received reduction and ten experienced spontaneous abortion of one fetus).

In multivariate logistic regression analysis, after adjusting for female age, female BMI, percentage of transferred blastocysts, number of embryos transferred per ET cycle, diagnosis of PCOS and freeze-all cycles, number of transferable embryos and AFC were both significant independent factors predicting cumulative live birth in a two-year period (p<0.001) (**Table 3**).

**Table 3:** Multivariate logistic regression analysis for the prediction of 2-year cumulative live birth of women aged 35 or older in IVF/ICSI cycles.

	B	Standard error	P value	Exp (B) (95% confidence interval)
Female age	-0.215	0.044	<0.001*	0.806 (0.741,0.878)
Number of embryos transferred per ET cycle	-0.162	0.183	0.375	0.850 (0.594,1.217)
Female body mass index	0.008	0.026	0.762	1.008 (0.957,1.062)
Diagnosis of PCOS	0.339	0.224	0.130	1.403 (0.905,2.175)
Number of transferable embryos	0.109	0.109	<0.001*	1.116 (1.074,1.158)
Antral follicle counts	0.022	0.011	0.045*	1.022 (1.001,1.044)
Percentage of transferred blastocysts	-0.374	0.376	0.321	0.688 (0.329,1.440)
Freeze-all cycle	-0.215	0.184	0.498	0.883 (0.615,1.267)

*Statistically significant.

## 4 Discussion

Results of this study indicated that, compared with age- and BMI-matched tubal factor controls, women with PCOS ≥ 35 years of age maintained better ovarian reserve and response, as well as prominently higher cumulative pregnancy rate and CLBR over two years in IVF/ICSI cycles.

The natural fecundity of women with PCOS was evidenced by the fact that those with both oligo-amenorrhea and hirsutism delivered at least one child as often as non-symptomatic women in a large-scale cohort study (17, 18). Women with PCOS and advanced age who achieve spontaneous pregnancy have also been reported (19). This may be attributed to a relatively good ovarian reserve, despite ageing.

Due to excessive initial recruitment and prolonged survival of follicles (6, 20, 21), polycystic ovaries exhibit a higher density and larger number of follicles compared with normal ovaries (6). In addition, biomarkers of ovarian reserve in women with PCOS are stable, regardless of age. A long-term follow-up study revealed that serum AMH levels and AFC were significantly higher in women with PCOS than in non-PCOS controls, and differences remained beyond 35 years of age, which accords with a lower serum FSH level because rising FSH is considered to be a hormone marker of menopause (9, 10). The decline in AFC in women with PCOS was significantly slower than that in controls (22), and a model based on age-related decline of serum AMH demonstrated that the predicted age of menopause in women with PCOS extended, on average, two years beyond that of normo-ovulatory women (23). Our results also demonstrated that AMH level and AFC in PCOS women ≥ 35 years of age were significantly higher than in controls.

The advantage of ovarian reserve in aging women with PCOS may confer a considerable ovarian response. This was illustrated by the lower total Gn dose, higher E2 level on trigger day, more oocytes retrieved, and larger number of transferable embryos in PCOS women in our study. Adequate oocytes retrieved provides considerable embryos for selection, resulting in pregnancy and LBR. This is consistent with other studies investigating IVF outcomes in those with PCOS and advancing age (12, 13, 24). Mellembakken et al. reported that oocyte count and LBRs in IVF/ICSI remained stable in women with PCOS who were 22-41 years of age, while those in the eumenorrheic comparison group decreased significantly with age (12). In the largest retrospective cohort study, Kalra et al. reported that women with PCOS demonstrated an approximately 20%–30% reproductive advantage in oocytes retrieved, pregnancy rate and LBR over women with tubal factor infertility until 40 years of age (13). However, pregnancy and LBR did not differ for each year after 40 years of age in the two groups. Hwang et al. reported that, when compared in subgroups according to age, pregnancy and LBR remained stable in women with PCOS until 38 years of age, while parameters declined significantly with age in tubal factor controls (24). Results of our study also revealed that women with PCOS exhibited significantly higher clinical pregnancy and LBR than controls in fresh ET cycles (clinical pregnancy rate per ET: 106/189[56.08%] in the PCOS group *vs*. 129/369[34.96%] in the control group, p<0.001; LBR per ET: 84/189[44.44%] in the PCOS group *vs*. 88/369[23.89%] in the control group, p<0.001). However, the values of AMH level and AFC in predicting pregnancy outcomes of aged women in IVF cycles remain controversial. Zhang et al. investigated IVF outcomes of women with discrepancies between age and AMH. Their study revealed that the LBR in the older high-AMH group was significantly higher than that in the older low-AMH group, and CLBRs were comparable between the young low-AMH group and the older high-AMH group (25). Dai et al. reported that in women ≥ 37 years of age, rates of implantation, spontaneous miscarriage and livebirth were similar between women with high AMH levels and those with low AMH levels (26).

The advantage of 60%-86% in pregnancy and live birth may, in part, be attributed to the high percentage of blastocysts transferred in fresh cycles in women with PCOS (55/386 [14.25%] in the PCOS group *vs*. 34/710 [4.79%] in the control group, p<0.001). A Cochrane review summarized low-quality evidence for live births and moderate for clinical pregnancy that fresh blastocyst stage transfer is associated with higher rates than fresh cleavage stage transfer (27). For PCOS patients with moderate risk for OHSS but not meeting the conditions for freeze-all strategy, it is possible that fresh ET would be delayed to D5 to determine whether ET should be canceled depending on the development of OHSS. Furthermore, an adequate number of oocytes retrieved played a major role as providing considerable embryos for both selection and blastocyst culture.

Theoretically, CLBR in aged PCOS patients could benefit from an abundance of oocytes retrieved providing considerable transferable embryos in subsequent frozen-thawed cycles. Paula et al. reported that the cumulative baby take-home rate did not differ between women with PCOS and non-PCOS controls undergoing their first IVF cycles (28). Li et al. compared the outcomes of women with PCOS and isolated polycystic ovaries (PCOs), with age-matched controls undergoing IVF treatment. The results indicated that women in the isolated PCO group, but not the PCOS group, had a significantly higher CLBR per cycle start compared with controls, even though both groups had more oocytes yielded and transferable embryos than controls (14). In addition, multivariate logistic regression analysis revealed that the total number of transferable embryos was the only significant factor predicting cumulative live births after adjusting for age and BMI, instead of the presence of PCOS or isolated PCO feature (14). A similar conclusion was drawn here, as the regression analysis in our study also revealed that the total number of transferable embryos and AFC were significant factors predicting cumulative live birth in two years, after adjusting for female age, BMI, percentage of transferred blastocysts, number of embryos transferred per ET cycle, diagnosis of PCOS and freeze-all cycles.

The discrepancies among studies may be due to the following reasons. First, the population differed across studies, and only individuals ≥ 35 years of age were included in our study. Large-scale retrospective and prospective randomized studies have illustrated that, in fresh IVF cycles, an increasing number of oocytes yielded improved pregnancy and live birth before reaching 15, after which the IVF outcomes worsened (29–31). In contrast, CLBR increased with oocyte number in unselected infertile patients, even after the number of oocytes exceeded 15 (32, 33). Therefore, cumulative live birth was expected in women with PCOS, due to favorable ovary reserve and resultant response, especially in aged women, whose age-matched counterparts experience significant follicle loss with age. Moreover, the fact that aged women with PCOS restore regular menses and experience spontaneous pregnancy indicates possible beneficial changes in fertility (34, 35). A study reported that AMH level and AFC could be used as prognostic indicators in a personalized prediction model of live birth (36).

Second, the heterogeneity of PCOS phenotypes may have different influences on outcomes. De Vos et al. reported that hyperandrogenic PCOS phenotypes have significantly lower CLBRs when compared with normoandrogenic phenotype D (polycystic ovary morphology+ ovulatory dysfunction) and individuals with isolated PCOM ovaries. However, phenotype D has a CLBR similar to that of isolated PCOM ovaries (37). He et al. reported that a subgroup with metabolic syndrome among PCOS infertile patients had fewer retrieved oocytes, fewer available embryos, and a lower oocyte utilization rate compared with PCOS patients without metabolic syndrome in IVF cycles (38). Further regression analysis revealed that the number of embryos transferred and the number of available embryos were positive; however, metabolic syndrome was negatively associated with CLBR. These results resemble hypotheses addressing the compromised oocyte quality of patients with PCOS.

Third, accumulating evidence supports significant and adverse effects of female obesity on IVF outcomes (39), and a similar conclusion has been drawn in the PCOS population (2). Because obesity and overweight are prevalent among individuals with PCOS, our study used PSM method to strictly control for confounding bias caused by both age and BMI (40), which may lead to different conclusions, while counterpart studies have only controlled for age.

Nevertheless, how oocyte quality is affected by aging and PCOS remains unclear. An increase in embryo aneuploidy, dysfunction of mitochondria and loss of cellular polarity are commonly observed in aged women, leading to a decline in oocyte quality and an increase in pregnancy loss in unselected populations (3). Advantages of ovarian reserve do not indicate corresponding oocyte quality in aged PCOS patients. In fact, studies have concluded that endocrine imbalance in PCOS populations, including FSH deficiency, hypersecretion of LH, and hyperandrogenemia, hyperinsulinemia have adverse effects on oocyte maturation, fertilization rates and embryo quality, consequently resulting in impaired pregnancy rates, and increased pregnancy loss (38, 41–43). Dysfunctions of glycerophospholipid metabolism and glycosphingolipid biosynthesis in the follicles of PCOS patients have been reported to be associated with declines in the two pronuclei fertilization rates during IVF procedures (44). Obesity and metabolic syndrome, common among PCOS patients, induce chronic inflammation of oocytes and elevated lipolysis in follicular fluid, thus compromising oocyte quality and ART outcomes in PCOS patients (38, 45–47). Sagvekar et al. also reported epigenetic dysregulation of genes involved in vital ovarian functions in patients with PCOS (48).

However, these results are controversial. A prospective, comparative study demonstrated that the rate of MII and morphologically abnormal oocytes, the percentage of top-quality embryos on day 3 were equivalent in both PCOM and control groups after ICSI (49). Studies have also suggested that PCOS is not associated with embryonic aneuploidy (50, 51). After comparing women with diminished ovarian reserve (age > 40 years), women with premature ovarian aging/occult primary ovarian insufficiency (age < 38 with abnormally low functional ovarian reserve (FOR) by age-specific FSH and/or AMH) and women < 38 years of age with normal ovarian reserve, Gleicher et al. concluded that low androgen levels are associated with diminished FOR at all ages (52). Qin et al. reported that basal T level was a predictor of ovarian response and pregnancy outcomes in women with diminished ovarian reserve (53). Long-term follow-up studies revealed that women with PCOS restored regular menses, experienced improvement in hormone imbalance, and achieved spontaneous pregnancy late in reproductive age (19, 24, 34, 35), which implies that aged women with PCOS may have better ovarian reserve and clinical outcomes undergoing IVF.

Our study was the first to explore CLBR among women with PCOS ≥ 35 year of age compared with age- and BMI-matched controls undergoing IVF/ICSI. However, one limitation of our study was its retrospective design. The percentage of blastocysts transferred can hardly be controlled in retrospective studies as a factor influencing pregnancy outcomes. Future prospective studies, however, may overcome this drawback and yield convincing results. Additionally, we failed to calculate CLBR after all frozen embryos were thawed and transplanted, because most PCOS patients have preserved embryos. In addition, some aged PCOS patients with minor subfertility may achieve pregnancy either spontaneously or with the simple treatment of ovulation induction or intrauterine insemination, as reported in previous studies (18). Selection bias may have influenced our results to some extent. Furthermore, parameters reflecting the quantity of ovarian reserve including AFC, number of retrieved oocytes and transferable embryos do not reflect the quality of oocytes and embryos. Moreover, our results did not include pregnancy complications, while PCOS was reported to be associated with gestational diabetes, pregnancy-induced hypertension, and premature delivery (54, 55).

In summary, the results of our study demonstrated that women with PCOS women ≥ 35 years of age had a higher CLBR over a two-year period compared with their age- and BMI-matched controls undergoing IVF/ICSI cycles. It will be interesting for further prospective studies to explore overall CLBR in aged PCOS patients, as well as to elucidate the underlying mechanisms.

## Data Availability Statement

The original contributions presented in the study are included in the article/supplementary material. Further inquiries can be directed to the corresponding authors.

## Ethics Statement

The studies involving human participants were reviewed and approved by Human Research and Ethics Committee of Sun Yat-sen Memorial Hospital, Sun Yat-sen University, Guangzhou, Guangdong, China. Written informed consent for participation was not required for this study in accordance with the national legislation and the institutional requirements.

## Author Contributions

ZM and ML conceived the study, extracted and analyzed the data. ZM and ML wrote the first draft of the manuscript which was revised by PP, LL, JH, XC, and DY. This study was supervised by XC and DY. All authors contributed to the article and approved the submitted version.

## Funding

This study was supported by the National Key Research and Development Program of China (2017YFC1001004 and 2016YFC1000205); National Natural Science Foundation of China (No. 81501224); the grants of Secondary Development Projects of Traditional Chinese Herbal Formula Compound (No. YZB20174002); the Special Fund of Chinese Medical Association for Clinical Research (No. 17020480717); the National Natural Science Youth Fund (No. 81703784); the Science and Technology Project of Guangdong Province (No. 2017A020213028); the 5010 grants of Sun Yat-Sen University (No. SYSU2014005).

## Conflict of Interest

The authors declare that the research was conducted in the absence of any commercial or financial relationships that could be construed as a potential conflict of interest.

## Publisher’s Note

All claims expressed in this article are solely those of the authors and do not necessarily represent those of their affiliated organizations, or those of the publisher, the editors and the reviewers. Any product that may be evaluated in this article, or claim that may be made by its manufacturer, is not guaranteed or endorsed by the publisher.
